# Attachment styles among students at the faculty of medicine of Sfax and suicidality

**DOI:** 10.1192/j.eurpsy.2025.1498

**Published:** 2025-08-26

**Authors:** Y. Yaich, A. Bouallegui, N. Ben Hamed, H. Khiari, A. Hakiri, G. Amri, R. Ghachem

**Affiliations:** 1 Psychiatry B, Razi hospital, Tunis, Tunisia

## Abstract

**Introduction:**

The theory of attachment is one of the most influential theories in developmental psychology. It was formulated by British psychologist John Bowlby in the 1950s and 1960s. This theory states that early relational experiences, particularly those with primary attachment figures (usually parents), influence the formation of internal working models that guide social interactions and attitudes toward oneself and others throughout life. The theory of attachment has important implications for adult relationships. An important time to study attachment is during university years, when many young adults are on their own for the first time and must establish new relationships which represents a moment of vulnerability.

**Objectives:**

The purpose of this study was to evaluate the prevalence of different attachment styles among medical externs using the RSQ scale and to examine the relationship between these attachment styles and suicidality.

**Methods:**

This is a cross-sectional, descriptive, and analytical study. It was conducted over a period of five months among students at the Faculty of Medicine of Sfax using an online form that included sociodemographic data, medical history, lifestyle habits and the “Relationship Scale Questionnaire” (RSQ)

**Results:**

The average age was 21.63 years. The distribution of students according to their attachment styles showed that avoidant attachment was the most prevalent (29%, n=150), and women exhibited more ambivalent attachment than men (p=0,031). Ambivalent attachment was significantly associated with sexual orientation (p=0,025).

Personal psychiatric history was associated with secure (p=0.008), ambivalent (p=0.005), and disorganized attachment styles (p=0.011). A significant link was found between personal history of suicide attempts and ambivalent attachment style (p=0.005).Our results were compared with existing literature and studies on this topic agree on the importance of assessing students’ attachment styles and their emotional needs.

**Conclusions:**

This study highlights the significant relationship between attachment styles and suicidality among students at the Faculty of Medicine of Sfax.

Further research is needed to advance our understanding of the implications of these findings and how to address the emotional and psychological needs of students.

**Disclosure of Interest:**

None Declared

